# Supplementary material for: Enhanced Intervertebral Disc Repair via Genetically Engineered Mesenchymal Stem Cells with Tetracycline Regulatory System
Source: Int J Mol Sci. 2023 Nov 7;24(22):16024. doi: 10.3390/ijms242216024 (PMC10671788; doi:10.3390/ijms242216024)
Supplement: Supplementary file 1 [file ijms-24-16024-s001.zip › ijms-2666234-supplementary.pdf]

International Journal of *Molecular Sciences*

MDPI

Supplementary Materials

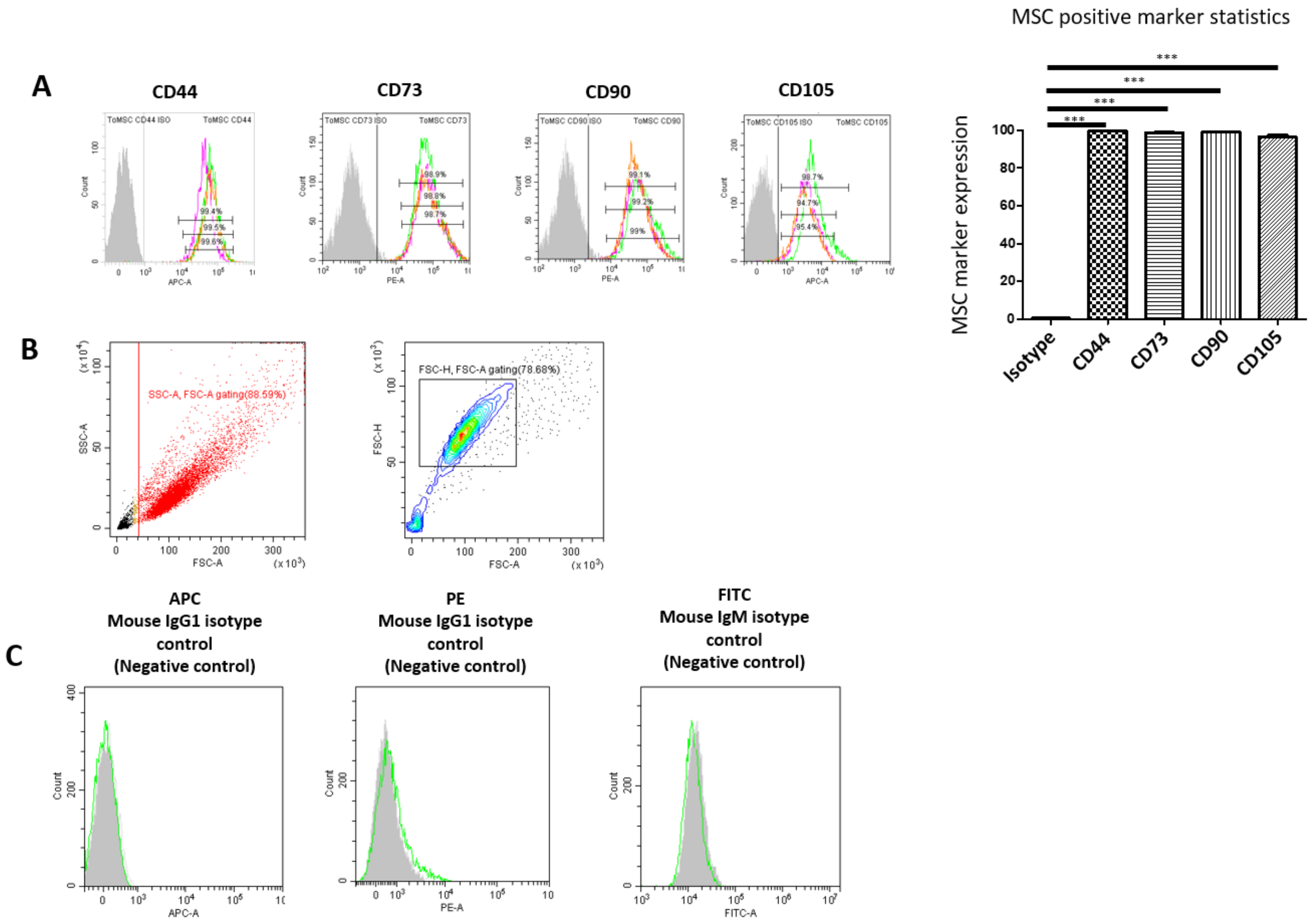

Figure S1. Flow cytometry statistical processing for ToMSCs and negative controls for antibodies APC, PE, FITC (A) Flow cytometry statistics MSC marker(CD44, CD73, CD90 and CD105). (B) Flow cytometry analysis gating (SSC-A, FSC-A) and (FSC-H, FSC-A). (C) Flow cytometry of analysis of negative control. . ***$p < 0.001$, indicating a significant difference between groups as determined by one-way analysis of variance.

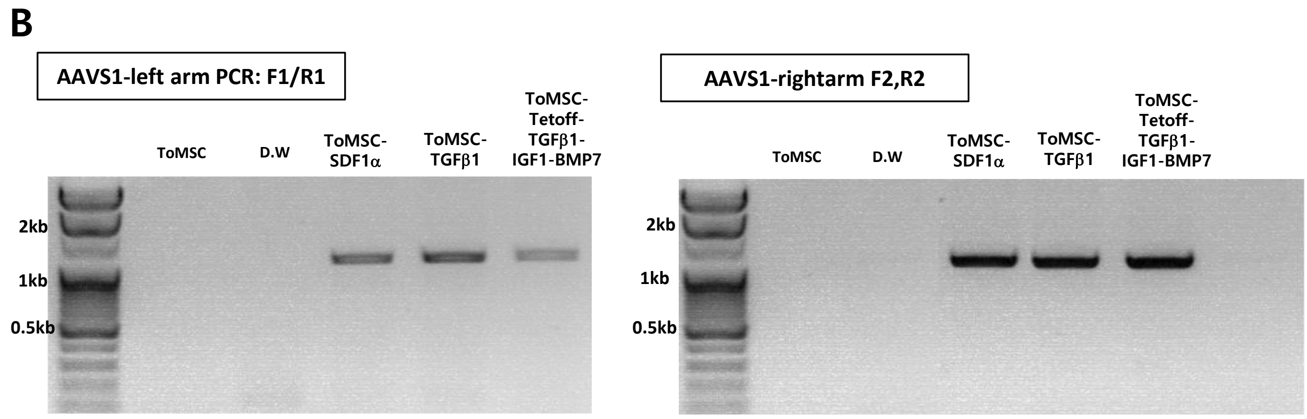

Figure S2. Uncropped gels image of Figure 2B. CRISPR/Cas9-mediated knock-in of transgenes into a safe-harbor site

(AAVS1) on the ToMSC chromosome.

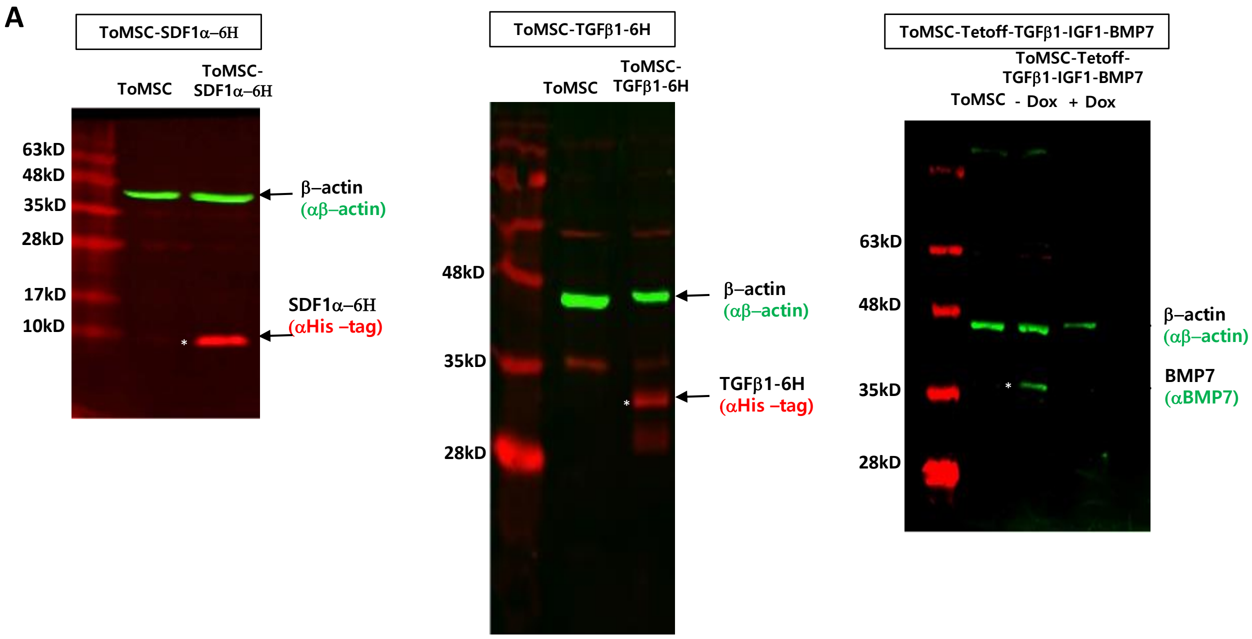

Figure S3. Uncropped blots image of figure 3A. Transgenic expression as evaluated by western blots, immunocytochemistry, and qRT-PCR.

Target	Primer sequence	Size	Annealing temperature (℃)	PCR cycle
AAVS1-left arm PCR	F1: 5′-CCT GAG TCC GGA CCA CTT TGA GC-3′<br>R1: 5′-GAG GAA GAG TTC TTG CAG CTC G-3	1,163 bp	63	30
AAVS1-right arm PCR	F2: 5′-AAC ATA CGA GCC GGA AGC AT-3′<br>R2: 5′-CGT TGT AAA ACG ACG GCC AG-3′	1,062 bp	63	30
TGFB1	F: 5′-CCC AGC ATC TGC AAA GCT C-3′<br>R: 5′-GTC AAT GTA CAG CTG CCG CA-3	148 bp	52.1	29
IGF1	F: 5′-TTG CTA AAT CTC ACT GTC ACT GC-3′<br>R: 5′-GCT CCG GAA GCA GCA CT-3′	173 bp	55.1	32
BMP7	F: 5′-GGG CTT CTC CTA CCC CTA CA-3′<br>R: 5′-ACG TCT CAT TGT CGA AGC GT-3′	163 bp	59.1	32
GAPDH	F: 5′-GGG GTG AAC CAT GAG AAG TAT GA-3′<br>R: 5′-GAG TCC TTC CAC GAT ACC AAA G-3′	153 bp	55.1	27

AAVS1-sgRNA	5′-CCCCACAGTGGGGCCACTAG<u>GGG</u>-3′	*PAM sequence was underlined.

Table S1. Sequences of PCR primers and sgRNA used in this study
